# Key recent advances in TB vaccine development and understanding of protective immune responses against Mycobacterium tuberculosis

**DOI:** 10.1016/j.smim.2020.101431

**Published:** 2020-08

**Authors:** Thomas J. Scriba, Mihai G. Netea, Ann M. Ginsberg

**Affiliations:** a South African Tuberculosis Vaccine Initiative, Institute of Infectious Disease and Molecular Medicine and Division of Immunology, Department of Pathology, University of Cape Town, Cape Town, South Africa; b Department of Internal Medicine and Radboud Center for Infectious Diseases, Radboud University Medical Centre, Nijmegen, Geert Grooteplein 8, 6525 GA Nijmegen, the Netherlands; c Department of Genomics & Immunoregulation, Life and Medical Sciences Institute (LIMES), University of Bonn, Germany; d Bill & Melinda Gates Foundation, Division of Global Health, Washington DC, United States

**Keywords:** Tuberculosis (TB), Vaccine(s), Adaptive immunity, Innate training, Correlates of protection

## Abstract

Tuberculosis is the leading infectious disease killer globally due to a single pathogen. Despite wide deployment of standard drug regimens, modern diagnostics and a vaccine (bacille Calmette Guerin, BCG), the global tuberculosis epidemic is inadequately controlled. Novel, effective vaccine(s) are a crucial element of the World Health Organization End TB Strategy. TB vaccine research and development has recently been catalysed by several factors, including a revised strategy focused first on preventing pulmonary TB in adolescents and adults who are the main source of transmission, and encouraging evaluations of novel efficacy endpoints. Renewed enthusiasm for TB vaccine research has also been stimulated by recent preclinical and clinical advancements. These include new insights into underlying protective immune responses, including potential roles for ‘trained’ innate immunity and Th1/Th17 CD4+ (and CD8+) T cells. The field has been further reinvigorated by two positive proof of concept efficacy trials: one evaluating a potential new use of BCG in preventing high risk populations from sustained *Mycobacterium tuberculosis* infection and the second evaluating a novel, adjuvanted, recombinant protein vaccine candidate (M72/AS01_E_) for prevention of disease in adults already infected. Fourteen additional candidates are currently in various phases of clinical evaluation and multiple approaches to next generation vaccines are in discovery and preclinical development. The two positive efficacy trials and recent studies in nonhuman primates have enabled the first opportunities to discover candidate vaccine-induced correlates of protection, an effort being undertaken by a broad research consortium.

## Highlights

• Highly efficacious TB vaccines would make a crucial impact in epidemic control.
• Elucidating mycobacteria-induced immune responses is expanding our understanding of vaccine-induced protection.
• Rigorous BCG revaccination trials are providing novel insights into TB vaccinology.
• A novel vaccine’s efficacy has launched TB vaccinology into the next wave of trials.
• Effort to identify nonhuman primate and human correlates of protection is advancing.

## 1 Introduction

### 1.1 TB global epidemiology

Even in the time of SARS-CoV-2, tuberculosis (TB) is to date the leading global infectious killer due to a single pathogen (i.e., the bacterium, *Mycobacterium tuberculosis* (Mtb)), and one of the world’s top ten causes of death. According to the World Health Organization [1], there were an estimated 10 million new cases and 1.4 million deaths due to TB in 2019. Substantial improvement in TB mortality rates, but only minimal global progress in decreasing TB incidence, have been achieved over the past 20 years [1]. COVID-19-related disruptions in TB services are predicted to cause a significant increase in global TB morbidity and mortality in coming years [[1], [2], [3]]. Compounding the urgency of the TB epidemic is the spreading challenge of drug resistant-TB: approximately 500,000 cases in 2019 of which 78% were multidrug-resistant [1].

### 1.2 The current vaccine for TB prevention: bacille Calmette-Guérin (BCG)

Reaching WHO’s End TB ambitious strategic goal of ending the TB epidemic by 2035 [4] will require effective vaccine(s) that can block the cycle of transmission. The only currently licensed vaccine to prevent TB is bacille Calmette-Guérin (BCG), which was first administered in Paris in 1921 by Dr. Benjamin Weill-Halle to a child, using the oral route [5]. BCG is still administered today (parenterally) to infants in most countries as part of the WHO’s Expanded Programme on Immunization. While infant BCG is moderately effective in preventing severe, extrapulmonary forms of TB in young children, it has had varied efficacy in preventing TB in adolescents and adults in multiple clinical trials [6], and has been ineffective in controlling the global epidemic.

### 1.3 Target populations and indications for novel TB vaccines to maximize public health impact

Adolescents and adults primarily develop pulmonary TB and are the leading drivers of TB transmission. Given the urgent need for novel, highly effective TB vaccines, these age groups have become the leading priority target population for TB vaccine development. Preventing TB among adolescents and adults will, by preventing transmission, help to protect all age groups most quickly. The same or other novel vaccines are also being developed, nonetheless, for other age groups. Additionally, the fastest global decreases in morbidity and mortality will result from implementing vaccine(s) that prevent TB when administered both before *Mycobacterium tuberculosis* (Mtb) (pre-infection vaccine) and after infection is already established but before active TB disease develops (post-infection vaccine). It has been estimated [7] that one quarter of the world population has been infected with Mtb. This represents a very large reservoir of potential cases of future active TB. The impact of each vaccine type in a given population will depend on whether the epidemic is primarily driven by recent infections or by reactivation of established, asymptomatic infections (typically referred to, although imprecisely, as latent TB infection or LTBI) [[8], [9], [10], [11]]. A pre-infection vaccine may initially be evaluated for a prevention of infection (POI) indication, but ultimately should be evaluated for its ability to prevent disease (POD), as this is the only TB endpoint clearly established to prevent morbidity and mortality. A third target indication, prevention of TB recurrence (POR) and/or as an adjunct to TB treatment is being pursued for some vaccine candidates. In the POR case, candidate vaccines are being evaluated for their ability to decrease rates of TB relapse and/or reinfection in TB patients after cure, which has been estimated at 2–8 % for standard treatment of drug-sensitive TB in various studies [[12], [13], [14], [15]]. Vaccines as adjuncts to TB treatment might increase cure rates, especially for MDR-TB or XDR-TB, and/or shorten duration of TB treatment, which is still a minimum of six months and typically up to two years for MDR and XDR-TB. The WHO has issued Preferred Product Characteristics for each of these types of novel TB vaccines [16,17].

### 1.4 The TB vaccine pipeline of clinical candidates

Currently, the global TB vaccine pipeline, to the best of our knowledge, includes 16 distinct candidates in various stages of clinical development, ranging from Phase 1 through Phase 3 (for definitions of phases of clinical development see [18]).

The global portfolio of clinical candidates is summarized and referenced in Table 1 and has been the subject of several recent reviews (for example, [19,20]), so only those candidates with published mid- or late-stage human efficacy data are discussed here.

**Table 1:** TB Vaccine Candidates in Clinical Development.

Vaccine Candidate	Phase	Platform	Efficacy Trial Endpoint or Target Indication#	Current Target Population(s) #	Vaccine Responsible Entity§	Clinical Trial Registry Number(s)^	Selected References
Ad5Ag85A	1	Subunit (viral vector) – human Adenovirus 5 expressing Ag85A			McMaster University	NCT00800670 NCT02337270	[82,83,84]

ChAdOx1	1	Subunit (viral vector) – chimp Adenovirus expressing Ag85A			University of Oxford	NCT01829490 NCT04121494 NCT03681860	[85,86]

GamTBvac	1	Subunit (protein-adjuvant) - Ag85A and ESAT6-CFP10 fusion with dextran-binding domain immobilized on dextran and mixed with an adjuvant consisting of DEAE-dextran core with CpG oligodeoxynucleotides			Health Ministry of the Russian Federation	NCT03255278 NCT03878004	[87]

AEC/BC02	1	Subunit (protein-adjuvant) – antigens: Ag85A and fusion protein of ESAT6/CFP10; adjuvant: BCG CpG and an aluminum salt-based adjuvant system			Anhui Zhifei Longcom	NCT03026972 NCT04239313	[88]

TB/FLU-04L	1	Subunit (viral vector) – replication-deficient influenza A vector expressing Ag85A and ESAT6	POD	Infants; Adolescents/Adults	Research Institute for Biological Safety Problems and the Research Institute on Influenza, Russia	NCT02501421	[89]

MTBVAC	2a	Live, attenuated Mtb -with deletions of *phoP* and *fadD26*	POD	Infants; LTBI(-) Adolescents/Adults	Biofabri	NCT02729571 NCT03536117 NCT02013245 NCT02933281	[90,91,92,93]

ID93+GLA-SE	2a	Subunit (protein-adjuvant) – fusion protein comprised of antigens: Rv1813, Rv2608, Rv3619 and Rv3620; adjuvant: TLR4 agonist glucopyranosyl lipid adjuvant (GLA) formulated in a squalene-in-water emulsion (SE)	POI, POR, Therapeutic	Adolescents/Adults; TB patients	IDRI, Quratis, Bio Farma PT	NCT03722472 NCT01927159 NCT02508376 NCT01599897 NCT03806699 NCT03806686 NCT02465216	[94,95,96,97] (Day et al., in press Lan Resp Med 2020)

RUTI	2a	Mycobacterial -inactivated (liposomal fragments of Mtb)	Therapeutic	Adult TB patients on drug treatment	Archivel Farma	NCT00546273 NCT01136161	[98,99,100]

M72/AS01_E_	2b	Subunit (protein-adjuvant) – fusion protein comprised of antigens: MTB32A and MTB39A; adjuvant: liposome-based MPL-A and QS21	POD	LTBI(+) Adolescents/Adults	GSK, Gates MRI	NCT00600782 NCT01262976 NCT00621322 NCT00950612 NCT00707967 NCT01098474 NCT00397943 NCT01669096 NCT01424501 NCT01755598 NCT02097095	[23,24,31,32,101,102,103,104,105,106,107]

H56:IC31	2b	Subunit (protein-adjuvant) – fusion protein comprised of antigens: ESAT6, Ag85B, and Rv2660; adjuvant: TLR9 agonist comprised of ODN1a and the antimicrobial peptide KLKL(5)KLK	POR	Adult cured TB patients	Statens Serum Institut	NCT01967134 NCT02378207 NCT02375698 NCT01865487 NCT02378207 NCT02503839 NCT03512249	[108,109,110]

DAR-901	2b	Mycobacterial - inactivated (*M.obuense*); intradermal administration	POI	LTBI(-) Adolescents	Dartmouth College	NCT02712424 NCT02063555	[25,111,112,113]

BCG revaccination	2b	Mycobacterial – live attenuated, licensed BCG Danish; intradermal administration	POI	LTBI(-) Adolescents	N/A	NCT02075203 NCT04152161	[22,109]

VPM1002	3	Mycobacterial - live attenuated (recombinant BCG); intradermal administration	POI, POD, POR	Infants, Children, Adolescents/Adults	Serum Institute of India, Vakzine Project Management GmbH	NCT00749034 NCT01113281 NCT01479972 NCT02391415 NCT03152903 NCT04351685∼ CTRI/2017/03/008266 CTRI/2019/01/017026	[114,115]

Immuvac	3	Mycobacterial -inactivated (Mw, *M.indicus pranii*) – heat-killed; intradermal administration	POD, Therapeutic	Children, Adolescents/Adults; Adult TB patients on drug treatment	MoST India; Cadila Pharmaceuticals	NCT00341328 NCT00265226* CTRI/2019/01/017026	[116]

Vaccae™*	3	Mycobacterial - inactivated (*M. vaccae*) formulated for intradermal injection	POD	LTBI(+) Adolescents/Adults	Anhui Zhifei Longcom	NCT01979900*	

V7	3	Mycobacterial - inactivated (*M. vaccae*); oral administration	Therapeutic	Adult TB patients on drug treatment	Immunitor LLC	NCT01380119 NCT01977768	[117,118,119]

§ Vaccine Responsible Entity is used here to denote the entity(ies) that own(s) the candidate and is(are) responsible for the overall development program of the vaccine candidate; this entity need not be the same as the Trial Sponsor (entity or individual with overall regulatory responsibility for a given trial).

^ Registry numbers of trials in process or completed.

∼ Not yet recruiting.

# As identified by Vaccine Responsible Party(ies).

+ Phase 2a trial reportedly in planning, but registry number not found in clinicaltrials.gov or in WHO ICTRP; no TBFLU-04 L primary publications identified in peer-reviewed literature through PubMed search at the time of this writing.

* Completed Phase 3 trial more than two years ago but results have not been published in peer-reviewed literature as of this writing to the best of our knowledge.

There is also a (frequently changing) number of candidates in discovery and preclinical stages of R&D. Two of these, CMV-TB and intravenous (i.v.) BCG, have been selected for discussion below because they have recently demonstrated unprecedented levels of protection in nonhuman primates and are spurring development of the next generation of novel candidates and discovery of potential vaccine-induced, immune correlates of protection.

Nonetheless, the TB vaccine R&D process remains hampered by insufficient global investment [21] and a lack of complete understanding of the human protective immune response to this complex pathogen and an absence of both validated animal models predictive of human vaccine efficacy and correlates of protection (see below). Despite the resulting requirement for a largely empiric vaccine development process, two positive proof of concept efficacy trials for TB vaccine candidates have recently been completed: one evaluating a potential new use of BCG – to protect a high-risk population from Mtb infection [see below; [22]], and one evaluating a novel adjuvanted recombinant protein vaccine candidate, M72/AS01_E_, to protect latently Mtb-infected adults from developing active TB disease [23,24]. Results from a recently completed POI efficacy trial in adolescents in Tanzania of a third candidate, DAR-901 (an inactivated form of the non-tuberculous mycobacterium, *M. obuense*), did not demonstrate statistically significant efficacy against either the primary or secondary trial endpoint [25]. The other 13 clinical candidates, as noted in Table 1, include additional mycobacterial candidates (both live attenuated and inactivated or lysates) and subunit candidates (virally vectored as well as adjuvanted recombinant protein candidates). Each of these candidates is currently being evaluated in the clinic for use in one or more age groups (infants, adolescents, adults) and for one or more target indications (prevention of Mtb infection, prevention of Mtb disease, prevention of TB disease recurrence or as an adjunct to TB treatment).

### 1.5 Recent results catalysing progress

#### 1.5.1 Interventional studies

Two positive proof-of-concept clinical efficacy trials of novel TB vaccine candidates were published in 2018–2019 and are currently galvanizing TB vaccine R&D. The first [23,24] was a Phase 2b trial evaluating the adjuvanted recombinant protein candidate, M72/AS01E, in LTBI+, HIV- adults (see section 1.5.1.1, below). The second [22] evaluated the ability of a novel subunit vaccine, H4:IC31, or BCG revaccination to prevent Mtb infection in a high risk of infection adolescent population (see section 1.5.1.2, below). These results, respectively, provide for the first time a catalyst to catapult a novel subunit TB vaccine candidate into late stage development for prevention of TB disease and to evaluate further a novel use of the hundred year old TB vaccine, BCG, for a potential policy recommendation to provide protection against Mtb infection in some high-risk populations.

##### 1.5.1.1 M72/AS01E proof of concept Phase 2b trial

M72/AS01_E_ is comprised of two Mtb antigens (Mtb32A and Mtb39A) in a recombinant fusion protein and the GSK proprietary adjuvant system, AS01, [[26], [27], [28], [29], [30], [31], [32]] also used in GSK’s Shingrix® vaccine and the malaria vaccine, RTS,S. In this randomized, double-blinded, controlled efficacy trial, 3575 Mtb-infected (Interferon Gamma Release Assay-positive (IGRA+)) adults in Africa without active TB or HIV infection were randomized 1 to 1 to receive either two doses of M72/AS01E or placebo, one month apart and followed for a total of three years for microbiologically confirmed active, pulmonary TB without evidence of HIV infection (primary endpoint). At the primary analysis, two years after the second vaccination, M72/AS01_E_ demonstrated 54.0 % vaccine efficacy (95 %CI: 2.9–78.2) [24]. At the final analysis, conducted after a median follow-up period of 2.7 years after the second vaccination, vaccine efficacy was 49.7 % (95 % CI: 2.1–74.2). The frequencies of severe adverse events, potentially immune-mediated diseases and deaths were similar between the two groups. Antibody and T cell responses were evaluated in an according-to-protocol subgroup of 244 participants (M72/AS01E - 120; placebo - 124). All participants in the M72/AS01E arm of this subcohort had IgG antibody responses to the M72 protein by month 2 and remained seropositive through month 36. Vaccine-induced, M72-specific CD4 T cells that co-expressed two or more cytokines, defined as “polypositive”, were observed in 23.5 % (95 %CI 12.8–37.5) of M72/AS01E vaccinees after the first vaccination and in 53.7 % (95 % CI 39.6–67.4) by month 36. These CD4 T cells expressed primarily interferon-γ, interleukin-2 or tumor necrosis factor-α or any combination of these cytokines. CD40L expression was low at all timepoints and CD8 T cell responses were not detected in any participants [23]. The large majority of trial participants provided biospecimens under informed consent for a substudy to discover potential correlates of risk and protection, which was underway at the time of this writing. Of interest, a study of BCG prime vaccination followed by boosting with intramuscular M72/AS01_E_ in a rhesus macaque model [33] also evaluated vaccine-induced immune responses and protection. No vaccine efficacy against Mtb challenge above that induced by BCG alone was demonstrated. It is important to note that the rhesus macaque model used in this study has several important differences compared to the phase 2b trial, including that the trial participants were Mtb-infected at baseline (IGRA+) and had received BCG as infants (if at all) approximately 18–50 years before being vaccinated with M72/AS01E (or placebo). By comparison, the NHPs were Mtb-uninfected at the time of vaccination and M72/AS01E was administered 16 and 20 weeks post-BCG. Moreover, the challenge dose of Mtb (16–20 cfu) was administered to the animals via bronchoscope. With this methodology, typically all unvaccinated animals become infected with Mtb and develop active TB disease, whereas humans typically inhale a single infectious Mtb bacterium in the form of airborne droplet nuclei to become infected and it is estimated that only 5–10 % of infected humans develop active TB disease. As a result, it is perhaps not surprising that the NHP model did not replicate the human results. Whether this model will accurately, consistently and reproducibly predict efficacy for TB vaccine candidates that protect Mtb-uninfected humans remains to be determined. Only one such vaccine, BCG, exists to date and both human and NHP studies of BCG have produced variable results [34,35]. The lack of a validated animal model predictive of human vaccine efficacy remains an important impediment to TB vaccine development.

##### 1.5.1.2 Intradermal BCG efficacy trials

Two potential new uses of BCG are currently under active investigation in clinical efficacy trials. The first, as noted above, is based on initial evidence that BCG delivered to Mtb-uninfected adolescents who were BCG-vaccinated as infants and at high risk of becoming Mtb infected were moderately protected from infection by being revaccinated with BCG [22]. This randomized, controlled, partially blinded trial (designated C-040-404; NCT02075203) was designed to evaluate the ability of BCG revaccination or of a novel adjuvanted recombinant protein vaccine candidate, H4:IC31, to prevent initial or sustained Mtb infection, as indicated by conversion from negative to positive of an Mtb-specific IGRA – each compared to placebo (the trial was not powered to compare these two vaccine candidates to each other). The trial enrolled 990 adolescents in the Western Cape of South Africa randomized equally across the three arms. Neither candidate demonstrated statistically significant protection against the primary endpoint of initial IGRA conversion but BCG demonstrated 45.4 % (p = 0.03; 95 % CI = 6.4, 68.1) efficacy in preventing sustained conversion (secondary endpoint), interpreted as reflecting prevention of LTBI. H4:IC31 demonstrated only 30.5 % (p = 0.16; 95 % CI=-15.8, 58.3) vaccine efficacy and its further development was terminated. A follow-up study characterised the breadth, function and phenotype of innate and adaptive cellular responses induced by BCG revaccination in a subset of participants of the C-040-404 trial [36]. BCG revaccination increased the magnitude of CD4 T cell subsets that expressed Th1 cytokines or IL-22, and also modestly increased IFN-γ-producing NK cells. Thus, protection against sustained Mtb-infection conferred by BCG may be dependent on multiple immune cell subsets. A study that aims to identify immunological correlates of protection in samples collected from C-040-404 trial participants is now underway (see Section 2.3 and Table 2).

**Table 2:** Major hypothesized immune correlates of protection against TB.

Immune response	Assays that measure the response	Source of evidence	References
Magnitude of mycobacteria-specific Th1/Th17 CD4 T cell responses	Intracellular cytokine staining assay; scRNA-seq^1^	NHP studies of mucosal or intravenous BCG vaccination; granuloma analysis in NHP study of Mtb infection	[49,50,51]
Mycobacteria-specific mucosal IgA or IgG antibody response magnitude, subclass and avidity	Binding antibody multiplex assay, avidity index assay^2^	BCG-vaccinated infants; in vitro activity of serum and monoclonal antibodies against Mtb	[50,59,120,121,122]
Fc-mediated, functional antibody activities	“Systems serology” approach to measure Fc-associated biophysical characteristics of mycobacteria-specific antibodies; antibody glycosylation analyses, function^3^	Human TB disease vs Mtb infection; Human resisters vs Mtb infected individuals; in vitro activity of serum and monoclonal antibodies against Mtb	[123,124]
Trained innate immunity	Innate cell responses to non-specific stimulation; EpiTOF^4^; scATACseq^5^; MGIA^6^	Murine and human studies of BCG vaccination	[47,75,76,125,126]

1 Single cell RNA sequencing, for example using SeqWell technology [127].

2 Binding antibody multiplex assay, avidity index assay [128].

3 ADNKA, antibody-dependent natural killer cell activation; ADCP, antibody-dependent cellular phagocytosis; ADCD, antibody-dependent complement deposition; ADNP, antibody-dependent neutrophil phagocytosis; and ADDCP antibody-dependent dendritic cell phagocytosis [123,124].

4 EpiTOF, epigenetic landscape profiling using cytometry by time-of-flight [129].

5 Single cell ATAC-seq, assay for transposase accessible chromatin by sequencing [75].

6 MGIA, mycobacterial growth inhibition assay [76].

BCG revaccination is currently undergoing evaluation in a second, larger trial in IGRA-negative South African adolescents (1800 participants assigned 1:1 to either BCG or placebo) with sustained IGRA conversion as primary endpoint, to confirm and extend these results (NCT04152161). Because BCG is an already licensed, inexpensive intervention with a very long and robust safety record in immunocompetent individuals, BCG revaccination could represent a useful tool for controlling Mtb infection in populations with high risk of infection, if the ongoing trial successfully confirms vaccine efficacy. Safety in HIV-infected, immunocompromised adolescents and adults remains an open question needing further evaluation before widespread implementation of BCG revaccination in this population.

Three large, community-based trials had previously evaluated BCG revaccination in a large range of age groups without demonstrating substantial overall protective efficacy against active TB disease, with the exception of some limited sub-groups (younger children in one of two geographic regions in Brazil and an uninfected sub-cohort in the India trial) [[37], [38], [39]]. However, it is important to note that these trials had significant methodologic and design differences from the C-040-404 trial including that the majority of participants in these trials were individuals of unknown Mtb infection status and the efficacy endpoints were TB disease rather than prevention of sustained infection as indicated by IGRA. In addition, the role of BCG strain diversity remains unclear, although differences in microbiological and immunological properties between the strains have been suggested [40]. For example, a meta-analysis of randomized controlled BCG efficacy trials concluded that there was not strong evidence that BCG strain was associated with efficacy [6], while an analysis of pediatric TB incidence in Kazakhstan over a four-year period in which three different strains of BCG (from Japan, Serbia and Russia, respectively) were administered concluded that BCG source did impact effectiveness [41].

The second potential new use of BCG is based on the hypothesis that non-specific effects of BCG, through induction of trained innate immune responses, may provide some protection from severe heterologous respiratory infections, including COVID-19 disease (see below) [42,43]. A number of clinical efficacy trials are underway in multiple countries to test this hypothesis (see, for example, clinicaltrials.gov and WHO clinical trials registry).

#### 1.5.2 Key preclinical advances

##### 1.5.2.1 Intravenous BCG in mice and nonhuman primates

There has been a continuous interest to compare the effect of the BCG route of administration, with various studies suggesting that alternative routes to subcutaneous (s.c.) or the commonly used intradermal (i.d.) route are more effective. Both aerosol [44] or intravenous (i.v.) vaccinations [45] have been suggested to provide increased protection against TB. A series of more recent studies have expanded this work, and an increased research interest in i.v. administration of BCG has emerged. In mice, i.v. administration of BCG resulted in potent T-cell responses, although they were poorly correlated with efficacy of the vaccine [46]. A recent study on the other hand demonstrated long-term effects by i.v. BCG administration not only in the lymphoid cell lineage, but also in the myeloid lineage and the precursors in the bone marrow [47]. In addition, this study showed that the protective effect by myeloid cells against Mtb can be transferred to naïve mice by adoptive transfer. These murine studies have been recently complemented with investigations in non-human primates (NHP), that also suggest important advantages of i.v. administration. When comparing intradermal, intratracheal and intravenous administration of BCG in NHP, Sharpe and colleagues observed that the most effective protection against TB was achieved by i.v. administration [48]. This was also associated with the strongest induction of multifunctional CD4 T cells producing TNF and IFNg in the i.v.-vaccinated macaques, and subsequently lowest organ pathology. These data are supported by recent studies that also showed that macaques vaccinated i.v. with BCG displayed increased protection against TB, compared to animals vaccinated through i.d. or aerosol route [49]. Although these experiments do not allow ascertainment of the exact mechanisms of protection, primarily because the i.v route was so protective that it precluded identification of immune responses associated with levels of protection [49], they do provide very important hypotheses that can be tested. I.v BCG induced very high numbers and frequencies of antigen-specific, bronchoalveolar Th1/Th17 cytokine-expressing CD4 (and CD8) T cells. Antigen-specific Th1/Th17 cells in the lung were also associated with protection against Mtb in another NHP study that delivered BCG by bronchoscope into the airways [50], while an NHP study of Mtb infected macaques also identified this cell subset as correlating with the degree of Mtb control in granulomas [51]. These studies highlight antigen-specific Th1/Th17 cells as a promising candidate correlate of protection (CoP) (See section 2.2 and Table 2).

##### 1.5.2.2 CMV-TB in nonhuman primates

Another promising preclinical result was achieved with a rhesus macaque CMV-vectored recombinant protein candidate, rhCMV/TB, when evaluated for immunogenicity and protection in a low-dose Mtb rhesus challenge model [52]. Two independent challenge experiments were reported to demonstrate a decrease in combined extra-pulmonary and pulmonary Mtb infection and disease of 68% compared to unvaccinated controls after one year of follow-up post-first vaccination. The vaccine induced and maintained high frequencies of highly-differentiated, Mtb-specific circulating and tissue-resident CD4 and CD8 T cell responses, but an antibody response was not detected. Unprecedentedly for any other peripherally delivered TB vaccine candidate to date, 14 of 34 vaccinated animals (41%) had no detectable TB disease by CT scan or at necropsy (in the two studies combined) compared to zero of 17 unvaccinated controls following challenge with the highly virulent Erdman strain of Mtb. In ten of these RhCMV/TB-vaccinated animals, Mtb was undetectable in all tissues tested at necropsy. RhCMV/TB is currently in pre-IND development by Vir Biotechnology (Vir-2020).

#### 1.5.3 Cohort studies and biosignatures of TB risk and disease progression

Animal studies provide opportunities to manipulate the exact timing, dose and route of Mtb infection and allow access to relevant tissue sites such that investigation of the complex interactions and kinetics that define immunologic mechanisms of protection can be performed. The elegant studies in animal models discussed above thus provide critical insights into the immune cells, their phenotypes and functions that mediate protection against Mtb in a manner that is not possible in humans. These studies have also generated hypotheses about the immune responses that mediate protective immunity against Mtb infection or TB disease. However, it is clear that the immunopathogenesis of Mtb in humans is complex, extremely heterogenic and subject to a very large variety of environmental and biological factors to such a degree that exact recapitulation with experimental animal models is not possible. Clinical studies of human cohorts therefore provide key opportunities to study human immunopathogenesis of Mtb and can advance our understanding of mechanisms that mediate risk of disease as well as protective immunity. A number of clinical studies have followed human participants with Mtb infection or known exposure to Mtb for months or years to characterise immunological changes that represent correlates of risk of TB disease. Analysis of blood transcriptomes, plasma proteomes and cellular phenotypes have shown that Mtb-infected but asymptomatic individuals who ultimately develop disease present with elevated inflammation and type I/II IFN responses [[53], [54], [55], [56], [57], [58]], activation of the complement cascade [54] and T cell activation [54,59]. Other studies have applied intensive clinical, phenotypic and radiological examination to identify asymptomatic study participants with radiological or microbiological evidence of subclinical disease and found that such individuals presented with similar blood inflammatory signals [60,61]. These inflammatory signals are consistent with those typically observed during microbiologically confirmed, clinical TB disease [[62], [63], [64], [65]]. It is thus clear that progression from Mtb infection to active TB proceeds through at least two intermediate asymptomatic stages, namely minimal or “incipient” TB, characterised by elevated inflammatory signals in the absence of radiological or microbiological evidence of disease, and subclinical TB, characterised by the presence of either radiological or microbiological evidence of disease, or both [11].

Based on this premise, many investigators have developed concise transcriptomic or proteomic signatures, or correlates of risk that can identify individuals with incipient or subclinical TB [53,[55], [56], [57], [58],^66^]. These tests may allow exclusion of individuals from clinical trials (for example, because a prophylactic vaccine may not be able to protect someone who has already progressed to incipient or subclinical TB) or, conversely, may allow selective inclusion of those with incipient or subclinical TB (for example, to determine if a therapeutic vaccine can reverse disease progression or as an adjunct to therapy, accelerate therapy).

Other innovative and novel approaches to studying protective immunity and the immunopathogenesis of Mtb in humans are being explored in the context of experimental medicine studies. For example, a recent study of bronchoscopic instillation of live BCG or PPD into the lungs of healthy participants allowed assessment of cellular immune responses and changes in gene expression at the site of Mtb infection in humans [67]. Small phase I trials have also investigated safety and immunogenicity after aerosol administration of novel TB vaccine candidates [68,69]. Such small, intensive and carefully controlled experimental trials provide important opportunities to define the host immune response and inform vaccine design and development, and may ultimately lead to a human challenge model for TB [70]. However, such invasive interventions are not generally considered to be practical delivery platforms for mass vaccination campaigns against TB.

## 2 Advances in understanding protective immune responses

### 2.1 Trained innate immunity

Development of vaccines in the last half century or more has been based on induction of specific adaptive immune responses endowed with long-term memory. The concept behind this approach is to induce priming of antigen-specific naïve B and T cells and subsequently generation of memory B and T cells. These memory lymphocytes will initiate a rapid and robust immune response upon re-infection with the same pathogen, thus leading to long-lasting protection (sometimes for the entire life) against the target infection.

However, recent studies provide evidence that some types of vaccines, especially live attenuated ones, also induce a long-term improvement in the anti-microbial function of innate immune cells, and this effect can also contribute to protection from reinfection. The reprogramming of the innate immune cells (e.g. myeloid and NK-cells) has been termed ‘*trained immunity’,* and represents a *de facto* innate immune memory [71]. The induction of trained immunity by live attenuated vaccines induces an integration of immunological signals, metabolic rewiring of cell metabolism, and epigenetic reprogramming, all processes necessary to mediate the induction of improved innate immune responses [72].

Induction of trained immunity has been reported to be an important component of the biological effects of BCG vaccination, which induces epigenetic and metabolic rewiring of myeloid cells though an NOD2-dependent mechanism [73,74]. Moreover, recent studies have also demonstrated long-term functional and transcriptional reprogramming of myeloid cell progenitors in the bone marrow, which explains the long-term effect of BCG on circulating myeloid cells [47,75]. The increase in the anti-mycobacterial function of the myeloid cells such as monocytes and macrophages has been suggested to contribute to the beneficial effects of BCG against TB [76]. Not only myeloid cells, but also other innate immune cell populations such as NK cells undergo an increase in their function after BCG vaccination [77] and NK-memory responses have been correlated with BCG effects in humans [78]. It would thus be tempting to speculate that vaccines able to induce both innate and adaptive memory responses would be more efficient against TB. Of the novel TB vaccine candidates, MTBVAC also has been shown recently to be able to induce trained immunity [79], and evidence of its efficacy is eagerly anticipated. No additional data are available regarding the capacity of other TB vaccine candidates or relevant adjuvants to induce trained immunity, and additional future studies are warranted.

### 2.2 Approaches to discovering human correlates of protection against Mtb infection and TB disease

Publication of positive efficacy results from the proof-of-concept trials of BCG revaccination and M72/ASO1_E_ vaccination marked an important turning point from a somewhat negative undercurrent that characterised TB vaccine development until 2018 [80]. This negativity was amplified by results of the first, large phase IIb efficacy trial of a “new-generation vaccine candidate”, MVA85A, in infants published in 2013. The trial did not demonstrate statistically significant efficacy of MVA85A vaccination of previously BCG-vaccinated 4–6-month-old infants against Mtb infection or active TB [81]. Demonstration of protection by BCG revaccination or M72/ASO1_E_ vaccination thus provided the first opportunity to identify immunological correlates of protection (CoP). Crucially, a limited set of blood samples that were specifically earmarked for this purpose were collected and stored with informed consent in the BCG revaccination and M72/ASO1_E_ phase 2b trials and an international ‘TB Immune Correlate Program’ consortium, led by the Gates Medical Research Institute and supported by vaccine manufacturers, sponsors of the two phase 2b trials, key funding agencies and trial investigators, has been launched. This consortium is charged with identifying and executing a strategy to test a number of *a priori* hypotheses that are informed by existing knowledge and results from recent animal models and clinical studies (Table 2). The desire to identify immunological CoP for TB has been great for decades because measurement of an immunological outcome rather than accrual of clinical endpoints in large and expensive clinical trials could significantly accelerate vaccine development. It also has potential to reveal putative mechanisms of protection, which are likely to spark more rational design to improve the efficacy of next generation vaccines.

However, the relatively small number of participants that reached clinical endpoints (57 participants had sustained QFT conversion in the C-040-404 trial and 39 participants developed TB disease in the M72/ASO1_E_ trial) in the phase 2b proof-of-concept trials restrict statistical power of these CoP discovery approaches. This limitation emphasizes the importance of larger, follow-up studies to confirm and strengthen evidence of vaccine efficacy, while providing an opportunity to validate the CoP identified in the currently ongoing efforts.

## 3 Discussion and conclusions

These recent successes in TB vaccine research illustrate that deployment of a highly efficacious vaccine against TB is likely within this decade. It is critical that TB vaccine development accelerates towards phase 3 licensure trials with innovative designs and the necessary urgency. Crucially, identification of correlate(s) of protection could be hugely valuable in speeding optimization and an expansion of target populations for M72/AS01E and also in streamlining triage and evaluation of next generation candidates. The remarkable “warp-speed” at which COVID-19 vaccines are being developed demonstrates what real urgency can achieve and serves as a benchmark for the TB field. Given the morbidity and mortality that is suffered globally due to TB, it is time to accelerate commitment, investment and implementation to stop the infectious disease that has killed the most human beings.

## Funding

This review article did not receive any specific grant from funding agencies in the public, commercial, or not-for-profit sectors. The authors received salary support as follows: AMG – Bill & Melinda Gates Foundation; MGN – supported by an ERC Advanced Grant (#833247) and a Spinoza grant of the Netherlands Organization for Scientific Research; TJS – supported from research grants to University of Cape Town from South African Medical Research Council, Bill & Melinda Gates Foundation, National Institutes of Health, European and Developing Countries Clinical Trials Partnership.

## Declaration of Competing Interest

Mihai G. Netea – declares patent applications/registration of nanobiologics to stimulate or inhibit trained immunity, and he is a scientific founder of Trained Therapeutix and Discovery; Thomas Scriba – declares patent applications/registrations of transcriptomic and proteomic biosignatures of TB risk and disease progression and research grants to University of Cape Town from South African Medical Research Council, Bill & Melinda Gates Foundation, National Institutes of Health, European and Developing Countries Clinical Trials Partnership; Ann Ginsberg - none
